# Surface integrity optimization for ball-end hard milling of AISI D2 steel based on response surface methodology

**DOI:** 10.1371/journal.pone.0290760

**Published:** 2023-08-25

**Authors:** Weimin Huang, Cong Wan, Guijie Wang, Guosong Zhang

**Affiliations:** College of Mechanical and Electronic Engineering, Shandong University of Science and Technology, Qingdao, China; University of Vigo, SPAIN

## Abstract

This study focuses on systematically revealing how cutting parameters influence the surface integrity of ball-end hard milled surface of AISI D2 steel and proposing optimization scheme from surface integrity, wear resistance and fatigue resistance perspective based on response surface methodology respectively. Results can be summarized into three aspects. Firstly, radial depth of cut with percent contribution ratio (PCR) 62.05% has a decisive influence on surface roughness, followed by spindle speed 13.25% and feed per tooth 6.63%. The work hardening degree was raised from 12.5% to 38.4% when spindle speed changed from 8000 rpm to 2000 rpm. Spindle speed and radial depth of cut are the most significant factor influencing residual stress. The PCR of spindle speed and radial depth of cut reached 73.47% and 18.63% for residual stress in feed direction, 47.11% and 37.51% in step-over direction, respectively. High residual compressive stress can be generated by lowering spindle speed and radial depth of cut benefiting from the aggravated squeeze between ball-end milling cutter and workpiece. Secondly, too small feed per tooth or too small radial depth of cut should be avoided from wear resistance point because though the surface microhardness can be improved, the surface quality will also be deteriorated. The combination of high spindle speed, small feed per tooth together with small radial depth of cut can meet the wear resistance and the machining efficiency requirement. Finally, a medium-sized cutting parameter combination should be adopted to realize satisfying material removal rate and fatigue resistance. This study can be used to guide the selection of cutting parameters during ball-end milling of hardened AISI D2 steel for dies/molds manufacturing industries.

## Introduction

In virtue of the good blends of hardness, toughness and strength, cold working steel AISI D2 is widely used for the manufacture of dies and molds, taking drawing dies, extrusion molds, and plastic injection molds for example [1]. This kind of material contains high amount of hard carbide particles and can maintain high strength and hardness under high temperature condition, which is beneficial for fatigue resistance and wear resistance. However, these characteristics are also responsible for high temperature, deteriorated surface and tendency to cutting tool wear generated in cutting process leading to hardened AISI D2 steel a typical hard-to-machine material [2].

In general, the traditional manufacturing process for dies and molds contains milling of AISI D2 steel in annealed condition, quenching and tempering treatments, electrical discharge machining, grinding and manual polishing [3, 4]. This is a time-consuming process and seriously affects the production efficiency and market competitiveness of enterprises [5]. With the rapid development of advanced coatings, cutting tool material and lubrication/cooling technology, the feasibility and advantage of applying hard milling technology to the die and mold manufacturing industry is becoming more and more obvious [6, 7]. This is because the application of hard milling technology can reduce manufacturing cost and shorten production period [8]. However, this process may introduce unsatisfactory surface integrity taking large surface roughness, tensile residual stress, and surface pits for example [9]. It is well known that the excessive wear and the fatigue spalling or fracture are the common typical failure modes for dies and molds. The failure mechanism is always closely correlated with the surface integrity. Hence, it is necessary to systematically reveal the relationship between cutting parameters and surface integrity during ball-end milling process of hardened AISI D2 steel. Furthermore, proposing optimum process scheme is also of great importance to improve the wear and fatigue resistance of dies and molds in actual production.

Many experimental studies have been performed to illustrate the machinability of hardened AISI D2 steel. Sahinoglu et al. [10] explored the machinability of hardened AISI S1 steel (60HRC) by hard turning process using CBN cutting inserts and found that feed rate has significant influence on surface roughness. Carreira et al. [11] performed hard milling experiments of hardened AISI D2 steel and concluded that the combination of high milling speed and small feed per tooth contributed to small surface roughness. However, the variation trends of surface microhardness and residual stress along with the cutting parameters was not obvious because of the rough surface. Kara et al. [12] investigated the influence of cutting parameters on the tool wear and surface roughness during dry cutting of hardened AISI D2 steel. They found that the coated cutting tool performed better compared with the uncoated cutting tool from surface roughness and tool wear standpoint. Abbas A T et al. [13] used an artificial neural network with Edgeworth-Pareto method to optimize the cutting parameter during face milling process of high-strength grade-H steel and established neural network models for different surface roughness and unit-volume machining time requirements. Markopoulos A P et al. [14] conducted face milling tests of hardened cold work steel AISI O1 and revealed the influence of cutting parameters (depth of cut, cutting speed and feed rate) on surface roughness, cutting forces, cutting power and machining cost based on Taguchi design of experiments method. Aqib et al. [15] compared the influence of minimum quantity lubrication (MQL) and nanofluids-based MQL (NFMQL) on cutting temperature and surface roughness during face milling process of hardened AISI D2 steel based on response surface methodology. They found that NFMQL performed better in terms of the ability to reduce cutting temperature and surface roughness than MQL. Mac et al. [16] put forward a novel method to improve the prediction accuracy of cutting force and chip shrinkage coefficient for end milling of cold work steel SKD11 with the method of simulation and experiment.

The wear and fatigue resistance of machined surface is closely correlated with the surface integrity. Zheng et al. [17] studied the wear resistance of hardened Cr12MoV surfaces with quadrangular pits feature formed by ball-end milling process. They found that the smaller the size of pits feature is, the better the wear resistance will be. Carreira et al. [11] found that cermet inserts led to higher compressive residual stress and lower surface roughness compared with cemented tungsten carbide inserts in end milling process of hardened AISI D2 steel, which is beneficial for the improvement of adhesion characteristics. Tang et al. [18] investigated the influence of hardness on wear behavior of hardened D2 steel. They concluded that wear resistance was sensitive to hardness and the discrepancy in hardness is responsible for the difference in wear mechanisms. Gong et al. [19] investigated the influence of rolling times on the wear resistance of ultrasonic rolled surface of Cr12MoV steel. They found that the thickness of plastic deformation layer increased and the surface carbide decreased with the increase of rolling times. This variation can inhibit the crack initiation and propagation and then improve the wear resistance.

It is well known that the fatigue performance is related to work hardening and the state of residual stress for machined surfaces. Different processing techniques lead to different surface integrity and then the discrepancy in fatigue performance for the same surface. Turned surfaces have better fatigue resistance than those generated by EDM and electrochemical machining process because of a higher residual compressive stress [20, 21]. Jesus et al. [4] compared the fatigue resistance of AISI D2 steel subjected to electrical discharging machining (EDM) and conventional grinding techniques, respectively. They found that large dendritic primary carbides were generated after EDM process, which is detrimental to the fatigue resistance. In addition, the specimens machined by EDM process showed higher fatigue notch sensitivity than the ones machined by grinding process. Zhang et al. [22] studied the surface and subsurface characteristics and mechanical properties of ground surface of LPBF 304L stainless steel. The results showed that grinding process can significantly improve the surface roughness and grinding induced grain refinement can improve the fatigue resistance of the workpiece. Zhang et al. [23] compared the fatigue resistance of gear suffered four typical manufacturing processes: carburizing and grinding, shot peening, barrel finishing, and barrel finishing after shot peening. They found that compared with the carburized and ground process, the gear contact fatigue life of shot peening, barrel finishing, and barrel finishing after shot peening can significantly improve the surface roughness, residual stress gradient and hardened layer, thus improving the fatigue resistance of gear contact surfaces.

Taken together, the influence of cutting tool material, cutting parameters, coating types and lubrication methods on the machinability of hardened AISI D2 steel has been studied by many researchers from surface finish, tool life, cutting force and cutting temperature standpoint. The mainly findings are that the increase of cutting speed contributes to good surface finish and the coated carbide tool is competent for hard cutting. However, these reported studies are mainly focused on high speed hard turning and end/face milling process. Little attention was paid on the ball-end hard milling process of hardened AISI D2 steel. Moreover, though the influence of surface roughness, residual stress and hardness on the wear or fatigue performance has been reported by many literatures, the systematic investigations for the selection of cutting parameters from wear resistance and fatigue resistance perspective is scarcely found during ball-end hard milling process of hardened AISI D2 steel.

Hence, this study aims to systematically investigate the influence of cutting parameters on surface integrity, especially considering that the ball-end milling operation always take place at the end of dies or molds manufacturing process, and then put forward specific selection scheme of cutting parameters for specific surface integrity requirement, good wear resistance and satisfying fatigue resistance, respectively. The findings in this study can provide guidance for the selection of cutting parameters during ball-end milling of hardened AISI D2 steel and contribute to the high-efficiency and high-performance manufacturing of dies and molds.

## Experimental procedures

### Material preparation

**Fig 1:**
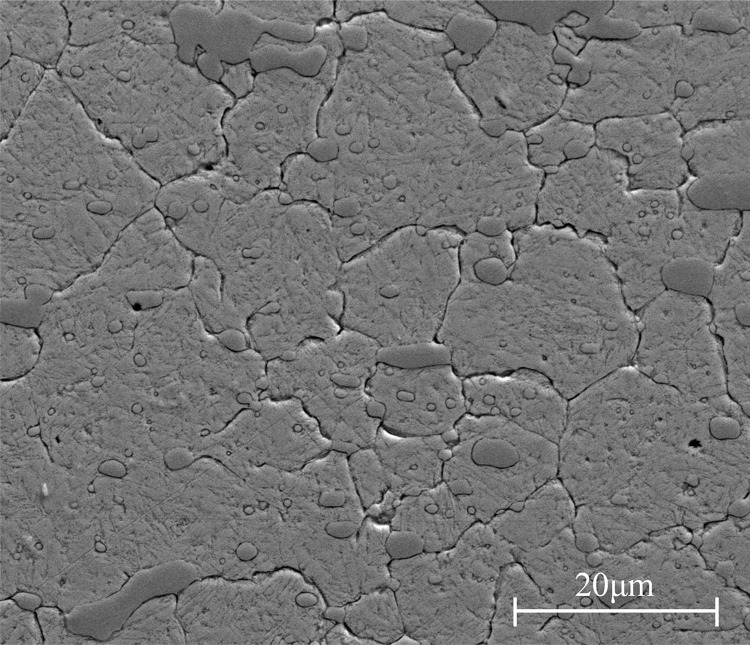
Microstructure of hardened AISI D2 steel.

The workpiece material selected in this study is high carbon and high chromium cold working steel AISI D2 with dimensions of 120 × 80 × 30 mm. In virtue of satisfying wear resistance, this kind of steel has been widely used in the dies and molds manufacturing industry. The chemical composition of the as-received AISI D2 steel is given in Table 1. After heat treatment, the Rockwell hardness of the used workpiece reached approximately 61±1 HRC. The substrate is composed of retained carbides, tempered martensite and retained austenite, as shown in Fig 1.

**Table 1:** Chemical compositions of AISI D2 tool steel.

Elements	C	Mn	Si	Cr	Mo	P	S	V	Fe
wt%	1.5	0.45	0.25	12	1.0	0.025	0.025	0.35	Balance

### Design of experiments based on response surface methodology

Response surface methodology (RSM) is usually used for modeling and analyzing the relationship between a response and several factors based on relatively small test numbers. A second-order mathematical model will be developed to realize the prediction of responses. This model considers not only the influence of independent factors but also the influence of interaction effect with each other. Response surface experiments were designed by using Box-behnken method. This method is commonly used during response surface design and does not arrange all test factors into a high-level test combination at the same time and then contributes to a satisfying cutting tool life. According to our preliminary experiments regarding of the relationship between cutting parameters and surface integrity, spindle speed *n*, feed per tooth *f*_z_, and radial depth of cut *a*_e_ were identified as the input factors. Three levels of the three selected factors are determined based on the existing research and the recommendation from the tool manufacturer. Furthermore, during the ball-end milling process, axial depth of cut was maintained a constant value 0.2 mm. 17 runs were designed based on the Design Expert 8.0 software. This software can provide highly efficient design of experiments and convenient response analysis. Table 2 shows the parameters configuration of the three factors and three levels response surface experiments designed based on Box-Behnken method.

**Table 2:** Parameters configuration of response surface experiments.

No.	Spindle speed	Cutting speed	Feed per tooth	Radial depth of cut *a*_e_, mm	Axial depth of cut *a*_p_, mm
	*n*, rpm	*v*_c_, m/min	*f*_z_, mm/z		
1	8000	251	0.30	0.3	0.2
2	8000	251	0.18	0.5	0.2
3	5000	157	0.18	0.3	0.2
4	8000	251	0.18	0.1	0.2
5	5000	157	0.18	0.3	0.2
6	2000	63	0.06	0.3	0.2
7	5000	157	0.30	0.1	0.2
8	5000	157	0.06	0.5	0.2
9	2000	63	0.18	0.5	0.2
10	5000	157	0.18	0.3	0.2
11	2000	63	0.30	0.3	0.2
12	5000	157	0.06	0.1	0.2
13	2000	63	0.18	0.1	0.2
14	8000	251	0.06	0.3	0.2
15	5000	157	0.30	0.5	0.2
16	5000	157	0.18	0.3	0.2
17	5000	157	0.18	0.3	0.2

**Fig 2:**
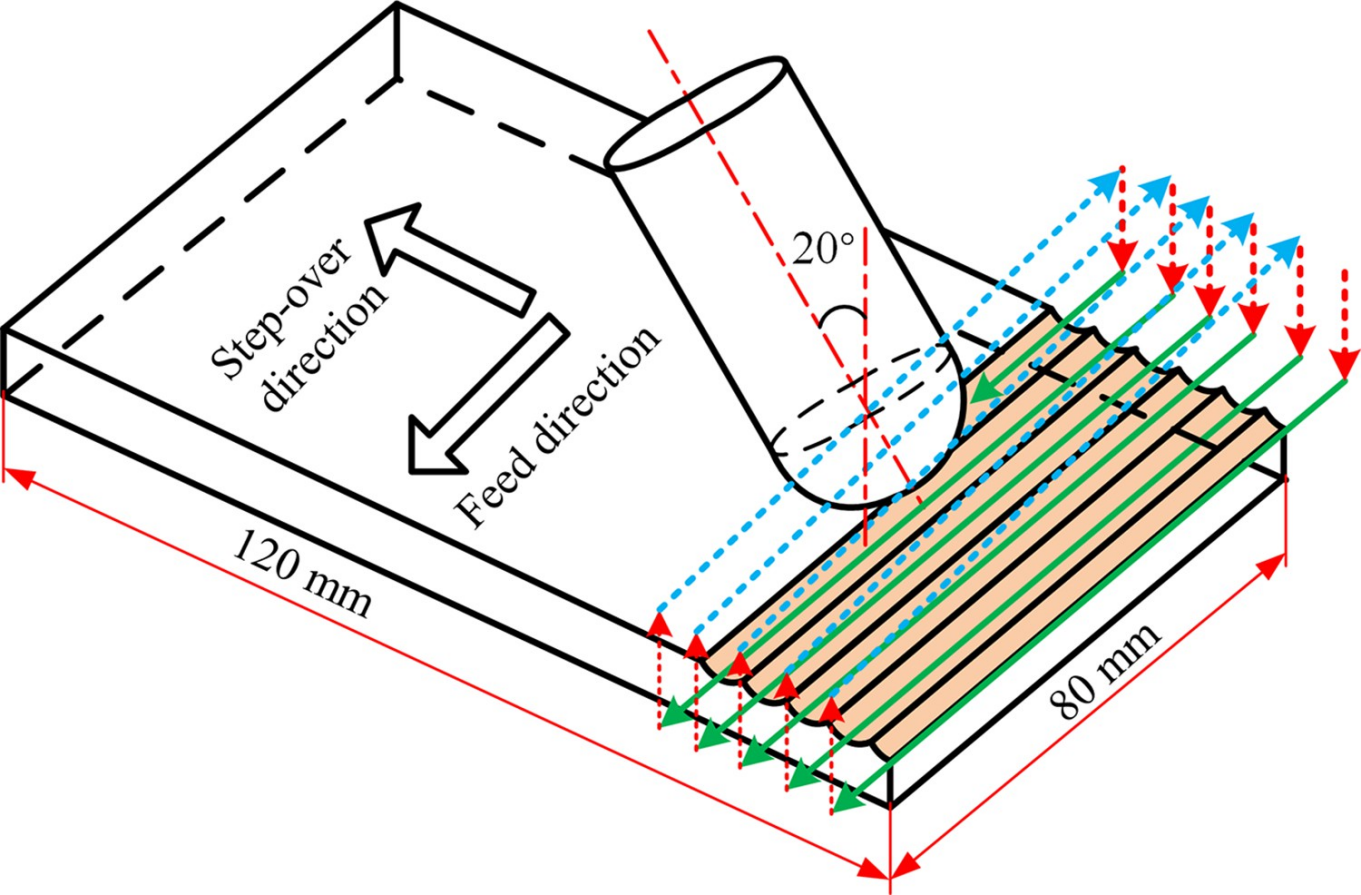
Schematic diagram of tool path strategy in ball-end hard milling process.

Then, the hard milling operations of AISI D2 tool steel were performed by using a five-axis high speed machining center DMU60P duoBlock. The maximum spindle speed of the machining center can reach 12000 rpm. New coated solid tungsten carbide ball-end milling cutter (Serial number SECO: TORNADO JH111L100-MEGA-64) was used during the unlubricated milling process. The geometrical parameter of the used ball-end milling cutter is given in Table 3. During the milling process, the lead angle *β*_f_ and the tilt angle *β*_n_ were set as 0° and 20°, respectively. According to previous studies [24, 25], this kind of combination can avoid the nose of ball-end milling cutter from cutting and contributes to a satisfying tool life. The schematic diagram of tool path strategy in ball-end hard milling process is shown in Fig 2.

**Table 3:** Geometrical parameter of ball-end milling cutter.

Diameter *ϕ*	Rake angle *γ*	Clearance angle *α*	Helix angle *β*	Tooth number *Z*
10 mm	0°	1°	17°	2

### Characterization of surface integrity

Before the characterization of surface integrity, the ball-end hard milled surface was cleaned carefully by using an ultrasonic cleaner for about 10 min to avoid the influence of oil contamination on detection accuracy. Surface roughness of milled surfaces was obtained by using white light interferometer Veeco NT9300. Three different areas on the ball-end milled surface were selected and the average value was used as the final surface roughness. Surface microhardness was measured through microhardness tester MH-6. The dwell time and the indentation load were set as 10 s and 0.1 kg, respectively. The singular results were eliminated and the microhardness was obtained by averaging at least five measurements. Residual stress was measured by using X-stress 3000 in the light of sin^2^*ψ* technique. Two directions, namely the feed direction and step-over direction, were adopted during the measuring process. At least five measurements were performed for each test and the average value was recorded as the final residual stress.

## Results and discussion

Surface roughness, surface microhardness, and surface residual stress were chosen as the three responses. The response surface analysis was conducted by using software Design Expert 8.0. The software can easily compare which polynomial model is the most suitable, and give the recommended model. This kind of software can compare different models and show the most significant one. By this way, the accuracy of the proposed prediction model can be improved to some extent.

### Response surface analysis for surface roughness

The analysis of variance (ANOVA) can be used to investigate the influence of input factors on output response [26]. Table 4 shows the result of variance analysis for surface roughness. Results show that spindle speed, radial depth of cut, feed per tooth and interaction between spindle speed and radial depth of cut are significant factors. The percent contribution ratio (PCR) on surface roughness reached 13.25%, 62.05%, 6.63% and 10.24%, respectively. Radial depth of cut is the most significant factor influencing surface roughness. The two factors interaction model was recommended for illustrating the results of surface roughness. It can be seen that the proposed model is significant as it exhibits a very small P value. The prediction model with R^2^ 93%, R^2^-adjusted 88.6% and R2-predicted 82.4% can be expressed by the following Eq (1).


$$\begin{array}{l} \mathrm{Ra}=0.305+1.17917\times 10^{-5}n+3.75521a_{e}-1.48175f_{z}-3.39167\times 10^{-4}na_{e}\\ +1.93056\times 10^{-4}nf_{z}-1.49479a_{e}f_{z} \end{array}$$ (1)

**Table 4:** Variance analysis for surface roughness: Two factor interaction model.

Source	Sum of squares	PCR(%)	df	Mean square	F value	P value Prob>F	Remarks
Model	1.54		6	0.26	21.75	< 0.0001	Significant
*A-n*	0.22	13.25	1	0.22	18.56	0.0015	
*B-a*_*e*_	1.03	62.05	1	1.03	86.72	< 0.0001	
*C-f*_*z*_	0.11	6.63	1	0.11	9.17	0.0127	
*AB*	0.17	10.24	1	0.17	14.01	0.0038	
AC	0.019	1.14	1	0.019	1.63	0.2301	
*BC*	5.15×10^−3^	0.31	1	5.15×10^−3^	0.44	0.5243	
Residual	0.12		10	0.012			
Lack of fit	0.039		6	6.58×10^−3^	0.33	0.8884	Not significant
Pure error	0.079		4	0.02			
Cor total	1.66		16				

**Fig 3:**
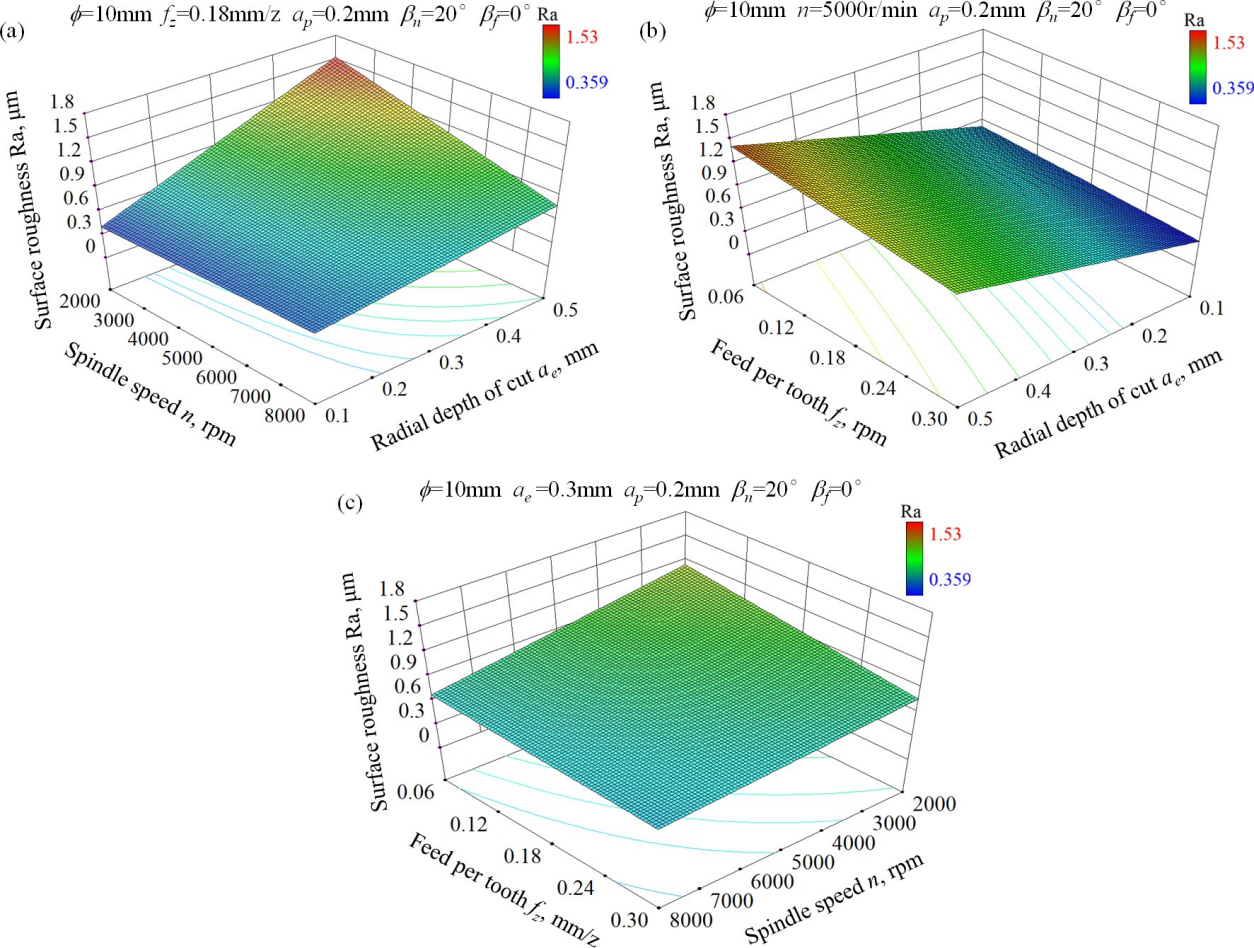
3-D response surface for surface roughness of ball-end milled AISI D2 steel under dry cutting condition, (a) *n*×*a*_*e*_, (b) *f*_*z*_×*a*_*e*_, (c) *f*_*z*_×*n*.

Fig 3 shows the 3-D response surface for surface roughness. Generally, surface roughness for ball-end milled surfaces mainly determined by two types of scallops: Feed-interval scallops induced by the feed movement and pick-interval scallops induced by the step-over movement. The difference in value between feed per tooth and radial depth of cut leads to the increase of heterogeneity of surface topography and then the increase of surface roughness. As indicated in Fig 3(A) and 3(B), radial depth of cut has the most significant influence on surface roughness compared with feed per tooth and spindle speed. This is because the pick-interval scallop height is usually higher than the feed-interval scallop. In addition, surface roughness decreases with the increase of spindle speed, but this effect is the least obvious. Though improving spindle speed contributes to the decrease of cutting force and the increase of cutting temperature, which is beneficial for cutting stability and surface roughness, this effect is negligible compared to the two types of scallops controlled by feed per tooth and radial depth of cut. Taken together, selecting small radial depth of cut and high spindle speed contributes to small roughness at given feed per tooth condition.

### Response surface analysis for microhardness

Analysis of variance for microhardness of ball-end hard milled surface is given in Table 5. Results show that spindle speed, radial depth of cut, feed per tooth, the interaction between spindle speed and radial depth of cut and quadratic terms of feed per tooth are significant factors. Feed per tooth (PCR 25.14%) and spindle speed (PCR 24.79%) are the most significant factor influencing surface microhardness relatively. The quadratic model was recommended for illustrating the results of surface microhardness. As shown in Table 5, the proposed model is significant because of the small P value 0.0037. The quadratic model with R^2^ value 92.3%, R^2^-adjusted 82.5% and R2-predicted 84.3% shown in Eq (2) can be used to describe the relationship between the three input factors and microhardness.


$$\begin{array}{l} \mathrm{HV}=1113.333-0.010167n-613.333a_{e}-613.888f_{z}+0.074167na_{e}-0.02nf_{z}\\ -708.33a_{e}f-2\times 10^{-6}n^{2}+462.5a_{e}^{2}+1770.8f_{z}^{2} \end{array}$$ (2)

**Table 5:** Variance analysis for microhardness: Quadratic model.

Source	Sum of squares	PCR(%)	df	Mean square	F value	P value Prob>F	Remarks
Model	38088.56		9	4232.06	9.39	0.0037	Significant
*A-n*	10224.5	24.79	1	10224.5	22.69	0.0021	
*B-a*_*e*_	2738	6.64	1	2738	6.08	0.0432	
*C-f*_*z*_	10368	25.14	1	10368	23.01	0.002	
*AB*	7921	19.21	1	7921	17.58	0.0041	
*AC*	256	0.62	1	256	0.57	0.4756	
*BC*	1156	2.8	1	1156	2.57	0.1533	
*A* ^*2*^	1364.21	3.31	1	1364.21	3.03	0.1254	
*B* ^*2*^	1441.05	3.49	1	1441.05	3.2	0.1169	
*C* ^*2*^	2737.89	6.64	1	2737.89	6.08	0.0432	
Residual	3154.5		7	450.64			
Lack of fit	3154.5		3	1051.5	0.15	0.9216	Not significant
Pure error	0		4	0			
Cor total	41243.06		16				

**Fig 4:**
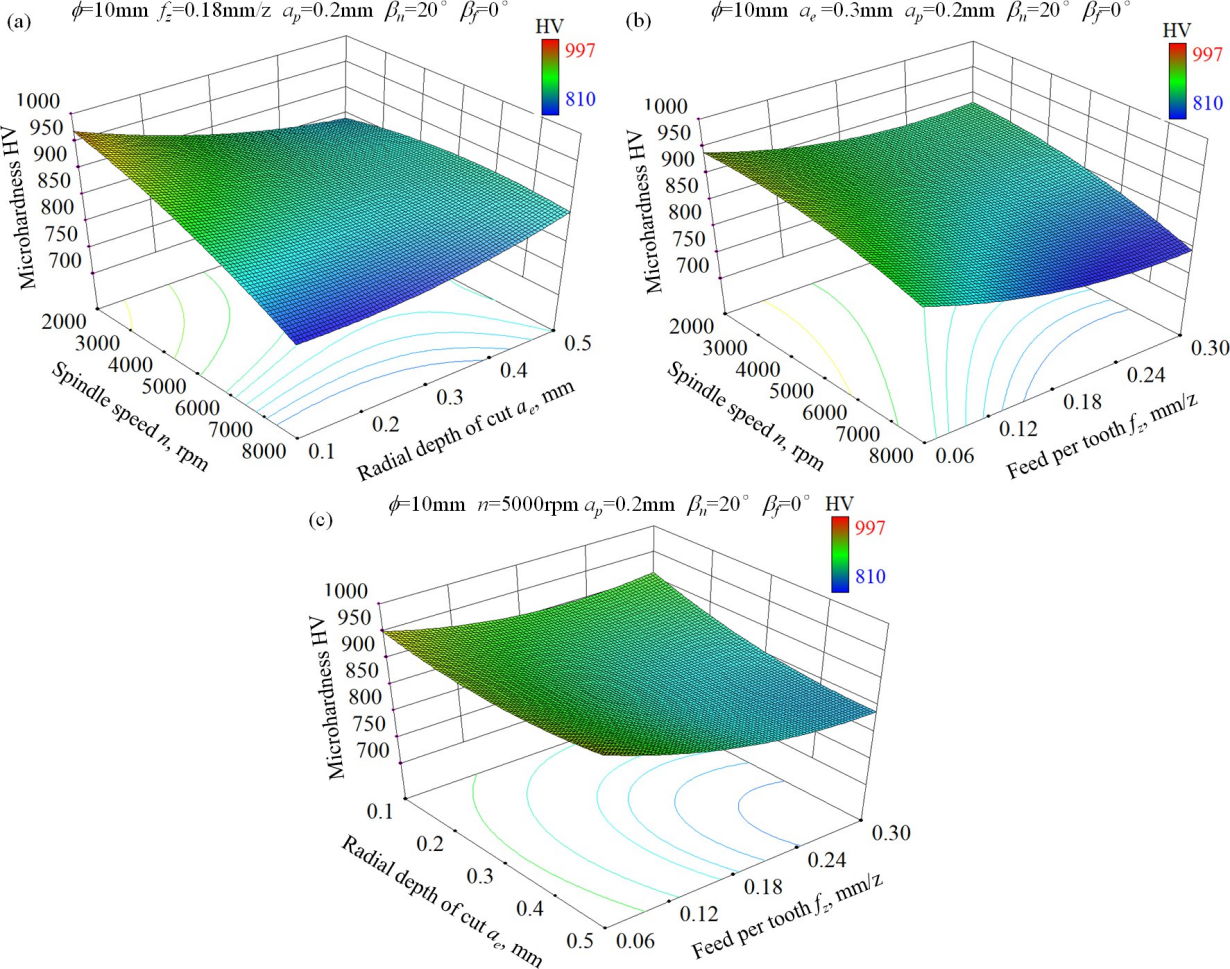
3-D response surface for microhardness of ball-end milled AISI D2 steel under dry cutting condition, (a) *n*×*a*_*e*_, (b) *f*_*z*_×*n*, (c) *f*_*z*_×*a*_*e*_.

Fig 4 depicts the 3-D response surface of microhardness considering the interaction effect of input factors. As a result of the coupling effect of strain hardening and thermal softening, obvious work hardening occurred after ball-end milling process of hardened AISI D2 steel, especially for the low spindle speed condition. Compared with the original hardness approximate 720 HV, the work hardening degree reached about 12.5% for the combination of *n* = 8000 rpm, *a*_*e*_ = 0.1mm, *f*_*z*_ = 0.18mm/z, and 38.4% for *n* = 2000 rpm, *a*_*e*_ = 0.1 mm and *f*_*z*_ = 0.18 mm/z condition. The reason can be explained from the following two aspects. On the one hand, due to the high hardness and high strength of hardened AISI D2 steel, a larger cutting force was needed compared with the traditional cutting process and lead to more severe plastic deformation. On the other hand, the good toughness of AISI D2 steel contributes to the accumulation of strain during hard milling process. As indicated in Fig 4, the microhardness of ball-end milled surface decrease with the increase of spindle speed and feed per tooth. This is because more heat was generated and the degree of thermal softening was strengthened under the circumstances. When a small spindle speed was adopted, microhardness of hard milled surface decreased with the increase of radial depth of cut due to the increased temperature. However, it is noteworthy that a slight increase of microhardness was presented for a high spindle speed condition. As spindle speed was set as 8000 rpm and feed per tooth 0.18 mm/z, the work hardening degree changed only from 12.9% to 15.7% when radial depth of cut increases from 0.1mm to 0.5mm. The reason for this is that though more heat was generated for the high spindle speed condition, the contact time between the tool flank face and machined surface was obviously reduced, and the cutting force was strengthened with radial depth of cut increase.

### Response surface analysis for residual stress

The variance analysis for residual stress in feed and step-over direction is shown in Tables 6 and 7, respectively. Results reveal that spindle speed, radial depth of cut and quadratic terms of feed per tooth are significant factors for residual stress in either direction. Furthermore, feed per tooth, the interaction between spindle speed and radial depth of cut, the interaction between spindle speed and feed per tooth and quadratic terms of spindle speed are also significant factors for residual stress in step-over direction. Overall, spindle speed and radial depth of cut are the most significant factor influencing residual stress. The PCR of spindle speed and radial depth of cut reached 73.47% and 18.63% for residual stress in feed direction, 47.11% and 37.51% for residual stress in step-over direction, respectively. The quadratic model was recommended for illustrating the results of residual stress. As shown in Tables 6 and 7, the proposed model is significant because of the very small P value. Eq (3) with R^2^ value 97.6%, R^2^-adjusted 94.6% and R2-predicted 96.3%, and Eq (4) with R^2^ value 99.8%, R^2^-adjusted 99.5% and R2-predicted 99.7% can be used to predict residual stress in feed direction and step-over direction, respectively.


$$\begin{array}{l} \sigma_{feed}=-1284.125+0.0928n+1674.375a_{e}+1751.041f_{z}+3.204\times 10^{-6}na_{e}-\\ 1.036\times 10^{-6}nf_{z}+2.458\times 10^{-6}n^{2}-553.125a_{e}^{2}-4487.847f_{z}^{2} \end{array}$$ (3)

$$\begin{array}{l} \sigma_{step-over}=-2162.771+0.18825n+4932.292a_{e}+2002.778f_{z}+0.14417na_{e}\\ -0.28681nf_{z}+770.8333a_{e}f_{z}-4.79167\times 10^{-6}n^{2}-6709.375a_{e}^{2}-1536.4583f_{z}^{2} \end{array}$$ (4)

**Table 6:** Variance analysis for residual stress in feed direction: Quadratic model.

Source	Sum of squares	PCR(%)	df	Mean squares	F value	P value Prob>F	Remarks
Model	1.32×10^6^		9	1.47×10^5^	32.29	< 0.0001	Significant
*A-n*	9.96×10^5^	73.47	1	9.96×10^5^	218.71	< 0.0001	
*B-a*_*e*_	2.52×10^5^	18.63	1	2.52×10^5^	55.45	0.0001	
*C-f*_*z*_	10296.13	0.76	1	10296.13	2.26	0.1763	
*AB*	4	0	1	4	8.79×10^−4^	0.9772	
*AC*	0	0	1	0	0	1	
*BC*	0.25	0	1	0.25	5.493×10^−5^	0.9943	
*A* ^*2*^	5180.02	0.38	1	5180.02	1.14	0.3215	
*B* ^*2*^	4960.87	0.37	1	4960.87	1.09	0.3312	
*C* ^*2*^	49864.76	3.68	1	49864.76	10.96	0.0129	
Residual	31860.95		7	4551.56			
Lack of fit	3.75		3	1.25	1.57×10^−4^	1	Not significant
Pure error	31857.2		4	7964.3			
Cor total	1.36×10^6^		16				

**Table 7:** Variance analysis for residual stress in step-over direction: Quadratic model.

Source	Sum of squares	PCR(%)	df	Mean squares	F value	P value Prob>F	Remarks
Model	2.66×10^6^		9	2.95×10^5^	415.52	< 0.0001	significant
*A-n*	1.25×10^6^	47.11	1	1.25×10^5^	1764.85	< 0.0001	
*B-a*_*e*_	9.98×10^5^	37.51	1	9.98×10^5^	1405.26	< 0.0001	
*C-f*_*z*_	7021.13	0.26	1	7021.13	9.88	0.0163	
*AB*	29929	1.12	1	29929	42.1	0.0003	
*AC*	42642.25	1.6	1	42642.25	60.03	0.0001	
*BC*	1369	0.05	1	1369	1.93	0.2077	
*A* ^*2*^	7830.59	0.03	1	7830.59	11.02	0.0128	
*B* ^*2*^	3.03×10^5^	11.4	1	3.03×10^5^	426.9	< 0.0001	
*C* ^*2*^	2061.12	0.08	1	2061.12	2.9	0.1323	
Residual	4972.75		7	710.39			
Lack of fit	6.75		3	2.25	1.81×10^−3^	0.9999	Not significant
Pure error	4966		4	1241.5			
Cor total	2.66×10^6^		16				

**Fig 5:**
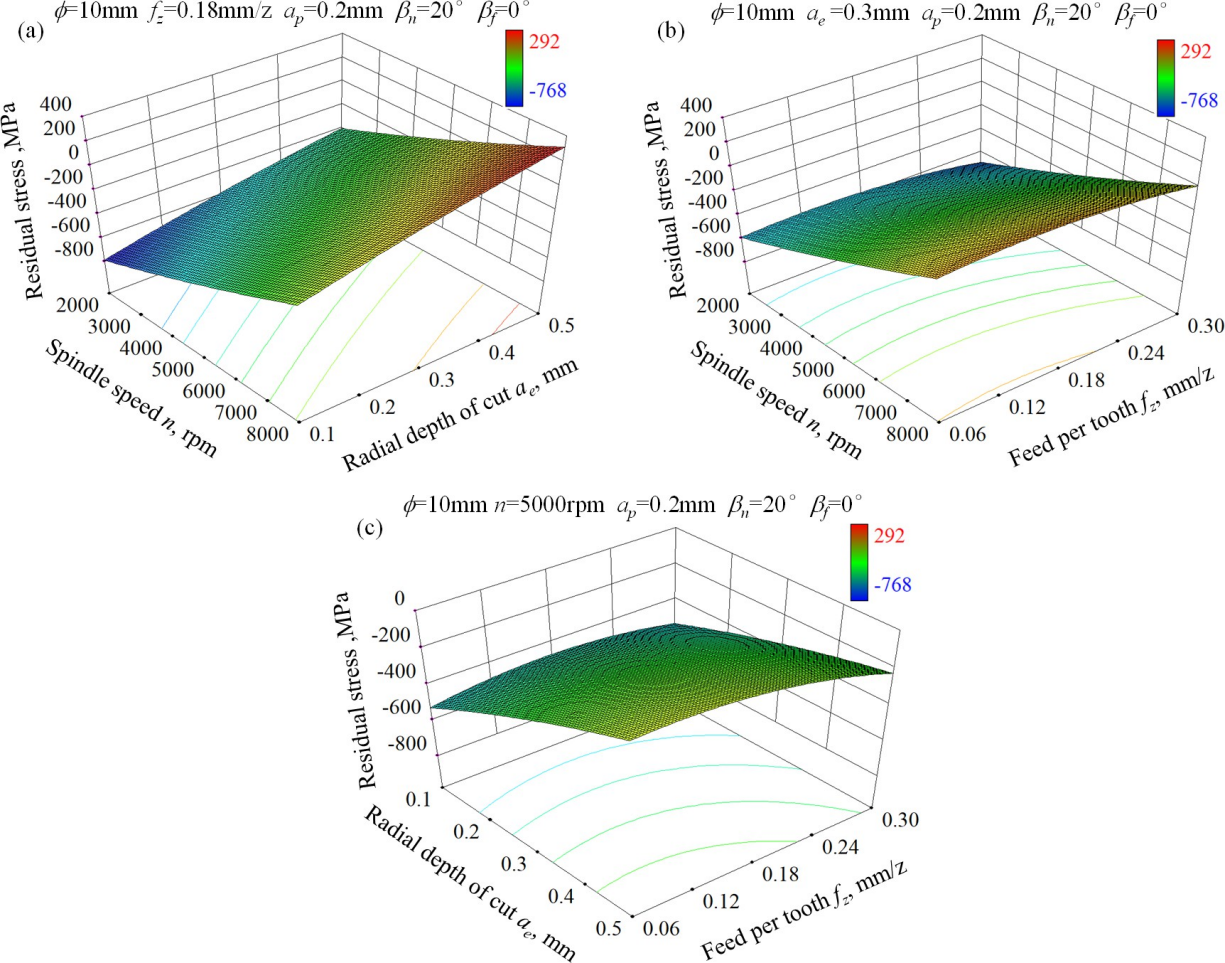
3-D response surface for residual stress in feed direction of ball-end milled AISI D2 steel under dry cutting condition, (a) *n*×*a*_e_, (b) *f*_z_×*n*, (c) *f*_z_×*a*_e_.

**Fig 6:**
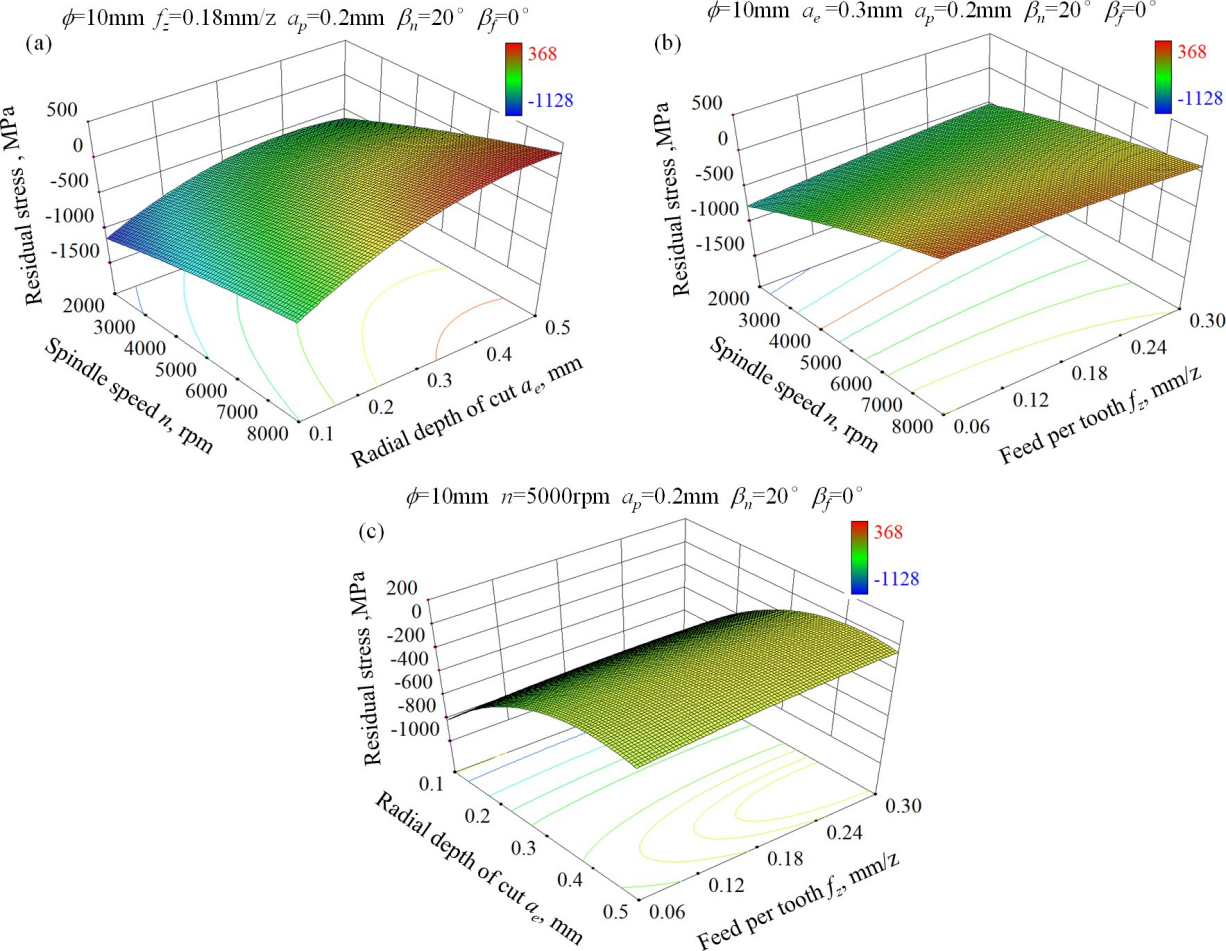
3-D response surface for residual stress in step-over direction of ball-end milled AISI D2 steel under dry cutting condition, (a) *n*×*a*_e_, (b) *f*_z_×*n*, (c) *f*_z_×*a*_e_.

Figs 5 and 6 show the 3-D response surface for residual stress in feed and step-over direction, respectively. It can be seen that spindle speed and radial depth of cut are the most notable influencing factors on surface residual stress among the three input factors. A combination of low spindle speed and low radial depth of cut contributes to high residual compressive stress because of the extended heat dissipation time and the aggravated squeeze between ball-end milling cutter and workpiece material. Conversely, the combination of high spindle speed and large radial depth of cut can bring about residual tensile stress due to the obviously increased cutting temperature. In addition, the effect of feed per tooth on residual stress in both directions is negligible especially at high spindle speed condition. Reasons for that can be explained as following. The thickness of chips changes continuously during the up milling process and reaches the maximum value as the exit of ball-end milling cutter from the workpiece surface. The heat accumulation also reaches the most severe level at this point. Due to the high spindle speed and the characteristics of intermittent cutting, most of heat is carried away by chips and little heat is diffused into the machined surface.

### Optimization of cutting parameters

According to the above analysis, the interaction of spindle speed and radial depth of cut has significant effect on surface integrity on the whole. Then the response surface of desirability function was established based on the two parameters aiming at specific goal of surface integrity, wear resistance and fatigue resistance, respectively.

#### Cutting parameters optimization for satisfying surface integrity

**Fig 7:**
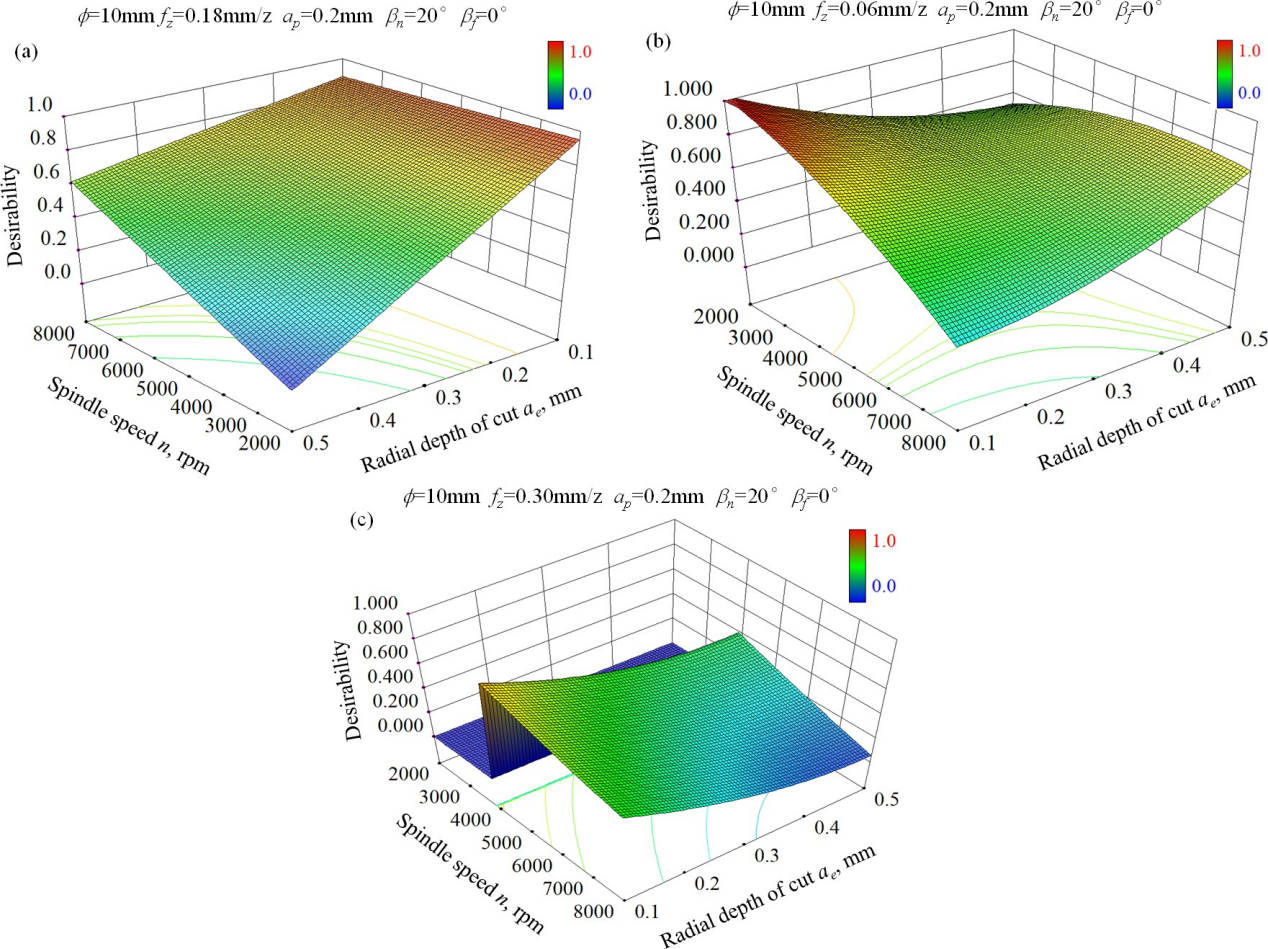
Response surface of desirability function for the goal of (a) Minimizing surface roughness, (b) Maximizing surface microhardness, and (c) Maximizing residual compressive stress.

Fig 7 depicts the response surface of desirability for different requirements of surface integrity. Two aspects can be summarized as following. On the one hand, desirability for minimizing surface roughness of ball-end milled surface is sensitive to radial depth of cut, especially for the low spindle speed condition as indicated in Fig 7(A). High desirability can be obtained when radial depth of cut is no larger than 0.2 mm. On the other hand, increasing spindle speed contributes to the improvement of desirability on the whole. When a small radial depth of cut is adopted, the desirability is basically maintained at a larger constant value. Then the combination of high spindle speed and small radial depth of cut can guarantee not only satisfying material removal rate but also high desirability for minimum surface roughness. The recommended cutting parameter configuration is given in Table 8. In actual production, the most reasonable parameter configuration should be selected comprehensively considering the cutting tool performance, the machine tool stability and the specific requirements of machined surface quality.

**Table 8:** Cutting parameter configuration for minimizing surface roughness with high desirability.

No.	Spindle speed *n*, rpm	Radial depth of cut *a*_e_, mm	Feed per tooth *f*_z_, mm/z	Ra	Desirability
1	4049	0.10	0.29	0.347	1
2	4548	0.10	0.30	0.353	1
3	4041	0.10	0.27	0.358	1
4	4238	0.10	0.29	0.357	1
5	4107	0.10	0.29	0.344	1
6	4002	0.10	0.28	0.355	1
7	4233	0.10	0.29	0.355	1
8	4105	0.11	0.30	0.351	1
9	4011	0.11	0.29	0.355	1
10	4393	0.10	0.30	0.349	1
11	4387	0.10	0.29	0.358	1
12	4097	0.10	0.29	0.352	1
13	4008	0.10	0.27	0.358	1
14	4440	0.10	0.30	0.351	1
15	4355	0.10	0.29	0.358	1
16	4145	0.11	0.30	0.349	1
17	4000	0.11	0.29	0.360	0.999
18	4000	0.12	0.30	0.365	0.995
19	4000	0.10	0.26	0.366	0.994
20	4990	0.10	0.30	0.368	0.992
21	4000	0.10	0.26	0.370	0.991
22	4000	0.10	0.23	0.391	0.973
23	5858	0.10	0.30	0.399	0.966
24	6428	0.10	0.30	0.420	0.948
25	6489	0.10	0.30	0.422	0.946
26	6779	0.10	0.30	0.432	0.937
27	5186	0.10	0.20	0.439	0.932
28	7199	0.10	0.30	0.447	0.925
29	7549	0.10	0.30	0.460	0.914
30	7567	0.10	0.30	0.460	0.913
31	7911	0.10	0.29	0.473	0.902
32	7981	0.10	0.30	0.475	0.901
33	7273	0.10	0.13	0.488	0.890
34	7868	0.10	0.13	0.491	0.887

Secondly, the desirability response surface is shown in Fig 7(B) for the goal of maximizing surface microhardness. It can be seen that there are two regions with relatively high desirability, namely the combination of small spindle speed and small radial depth of cut followed by the combination of large spindle speed and large radial depth of cut. However, for the later condition, large surface roughness and severe cutting tool wear will be introduced because of the increased cutting force and cutting temperature. For the former condition, though high desirability can be obtained, the material removal rate is too small to be suitable for mass production, but only for small batch production. Table 9 shows the recommended cutting parameter configuration for maximizing surface microhardness with high desirability. In actual production, the cutting parameter configuration should be determined taking production type into consideration.

**Table 9:** Cutting parameter configuration for maximizing surface microhardness with high desirability.

No.	Spindle speed *n*, rpm	Radial depth of cut *a*_e_, mm	Feed per tooth *f*_*z*_, mm/z	HV	Desirability
1	2091	0.10	0.07	1001	1
2	2041	0.10	0.07	1001	1
3	2287	0.10	0.06	1001	1
4	2067	0.10	0.06	1002	1
5	2116	0.11	0.06	1001	1
6	2013	0.10	0.06	1004	1
7	2109	0.10	0.06	1003	1
8	2037	0.11	0.06	1000	1
9	2226	0.10	0.06	1000	1
10	2013	0.10	0.07	1000	1
11	2054	0.10	0.07	1000	1
12	2000	0.10	0.08	998	0.991
13	2000	0.12	0.06	998	0.989
14	2588	0.10	0.06	998	0.989
15	2000	0.10	0.09	993	0.962
16	2000	0.10	0.1	990	0.945
17	2018	0.10	0.3	983	0.913
18	2000	0.10	0.3	983	0.908
19	2000	0.10	0.3	982	0.905
20	2237	0.10	0.3	980	0.892
21	3710	0.10	0.06	979	0.891
22	2000	0.19	0.06	973	0.859
23	2000	0.19	0.06	971	0.845
24	2000	0.10	0.21	968	0.832
25	6388	0.50	0.06	952	0.749
26	6434	0.50	0.06	952	0.749
27	6349	0.50	0.06	952	0.749
28	6201	0.50	0.06	952	0.749
29	6051	0.50	0.06	952	0.748
30	5330	0.50	0.06	950	0.737
31	5718	0.47	0.06	944	0.705
32	3611	0.50	0.06	937	0.668
33	4128	0.37	0.06	935	0.659
34	4140	0.38	0.06	935	0.659

Finally, it is well known that surface residual compressive stress contributes to fatigue resistance of service parts bearing alternate load. Fig 7(C) shows the desirability response surface for maximizing surface residual compressive stress in both feed direction and step-over direction simultaneously. It can be seen that the desirability gradually decreases with the increase of spindle speed no matter what radial depth of cut was used. Moreover, selecting a small radial depth of cut contributes to high desirability. The combination of small spindle speed and small radial depth of cut can bring about high desirability. However, this condition will seriously influence the material removal rate. It is noteworthy that the discrepancy in desirability is not obvious for surfaces ball-end milled with small radial depth of cut when different spindle speeds are used. Then selecting relatively high spindle speed and small radial depth of cut can guarantee not only high residual compressive stress but also a satisfying machining efficiency. Table 10 shows the recommended cutting parameter configuration for maximizing residual compressive stress with high desirability.

**Table 10:** Cutting parameter configuration for maximizing residual compressive stress with high desirability.

No.	Spindle speed *n*, rpm	Radial depth of cut *a*_*e*_, mm	Feed per tooth *f*_*z*_, mm/z	Residual stress *σ*_*feed*_, MPa	Residual stress *σ*_*cross-feed*_, MPa	Desirability
1	4000	0.10	0.06	-633	-952	0.884
2	4000	0.10	0.06	-630	-945	0.880
3	4000	0.11	0.06	-625	-925	0.871
4	4000	0.10	0.07	-610	-944	0.870
5	4131	0.10	0.06	-617	-933	0.870
6	4000	0.11	0.06	-620	-907	0.862
7	4337	0.10	0.06	-591	-903	0.848
8	4000	0.11	0.07	-593	-895	0.846
9	4000	0.10	0.10	-560	-924	0.840
10	4000	0.10	0.11	-540	-916	0.827
11	4000	0.10	0.30	-561	-861	0.819
12	4033	0.10	0.30	-557	-859	0.816
13	4000	0.10	0.13	-518	-905	0.813
14	4000	0.10	0.30	-556	-843	0.811
15	4000	0.10	0.28	-537	-862	0.808
16	4000	0.10	0.28	-535	-862	0.807
17	4157	0.10	0.30	-541	-849	0.806
18	4000	0.10	0.27	-526	-863	0.803
19	4000	0.10	0.26	-514	-864	0.797
20	4000	0.10	0.18	-488	-884	0.792
21	4000	0.10	0.24	-497	-867	0.791
22	4000	0.10	0.23	-492	-870	0.789
23	4000	0.16	0.06	-560	-718	0.770
24	5769	0.10	0.30	-351	-738	0.676
25	7863	0.10	0.30	-134	-630	0.525
26	4000	0.47	0.06	-294	-311	0.507

#### Cutting parameters optimization for wear resistance

**Fig 8:**
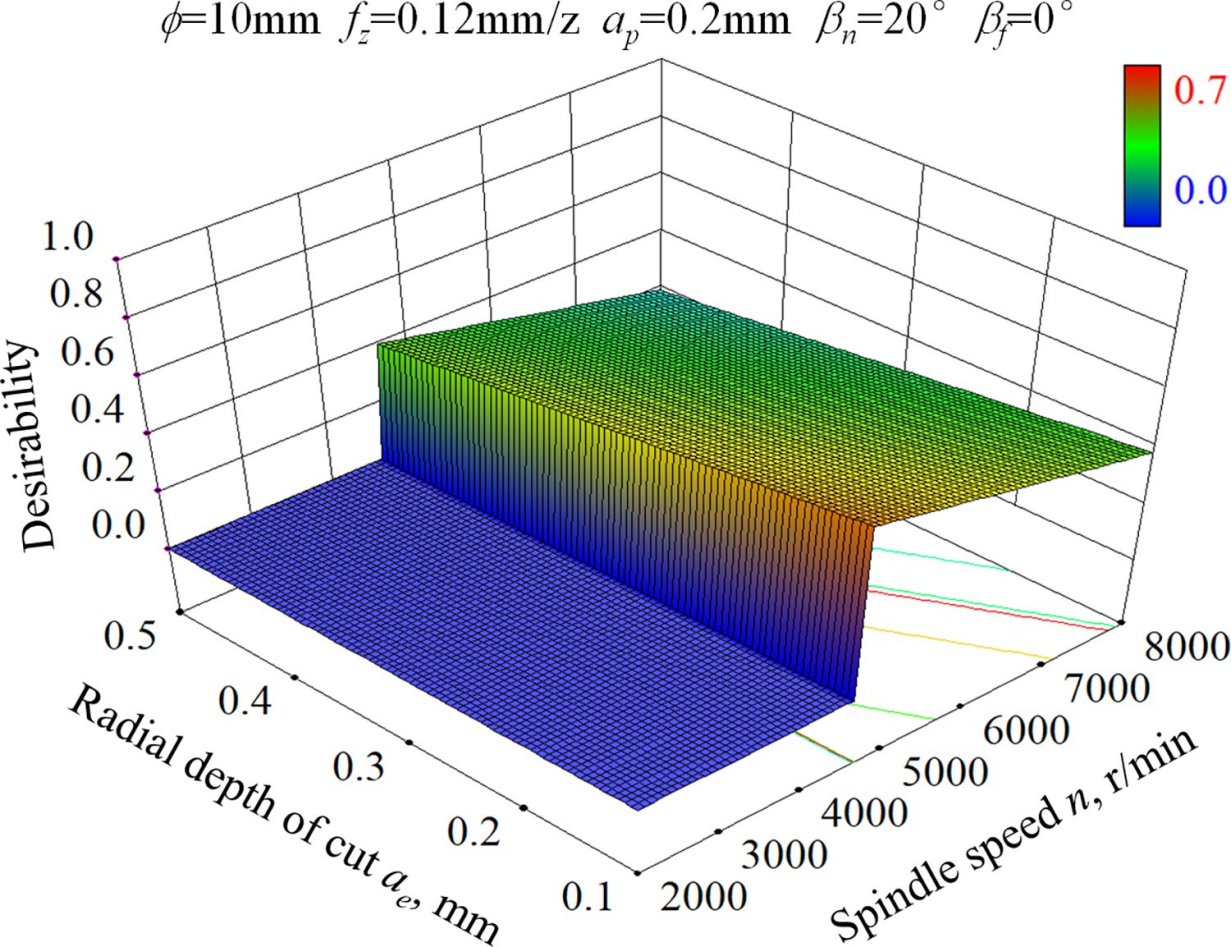
Desirability response surface for wear resistance of ball-end milled surface of AISI D2 steel under dry cutting condition.

According to our previous research, surface work hardening occurred during ball-end milling process of hardened AISI D2 steel contributes to the improvement of wear resistance. This is because work hardening can improve the ability to resist abrasive wear and plastic deformation on the surface, and also enhance the stability of the oxide film formed on the friction interface, thereby reducing the friction coefficient to a certain extent and improving wear resistance [1, 27]. Though the combination of small feed per tooth and small radial depth of cut is beneficial for high microhardness, the machining efficiency is greatly reduced. Furthermore, the surface quality will be deteriorated due to the occurrence of micro-pits defect caused by too small feed per tooth or too small radial depth of cut. Therefore, considering the above factors and the recommended cutting parameters of the tool manufacturer, two constraints are proposed as follows: 4500 rpm≤*n*≤8000 rpm, 0.12 mm/z≤*f*_*z*_≤0.3 mm/z. The desirability response surface for the goal of maximizing surface microhardness was obtained by Design Expert 8.0, as shown in Fig 8. The range of desirability is 0 to 1. It can be seen that the desirability decreases obviously with the increase of radial depth of cut and spindle speed. The satisfying solution with high desirability mainly exists in the condition of small radial depth of cut and low spindle speed. The optimization scheme of cutting parameters with high desirability for satisfying wear resistance is given in Table 11. In actual production, the cutting parameter combination with high desirability should be prioritized on condition that the ball-end milling cutter and machine tool performance meet the requirements.

**Table 11:** Cutting parameter configuration for wear resistance with high desirability.

No.	Spindle speed *n*, rpm	Feed per tooth *f*_*z*_, mm/z	Radial depth of cut *a*_*e*_, mm	HV	Desirability
1	4500	0.12	0.10	926	0.618
2	4500	0.12	0.11	925	0.614
3	4566	0.12	0.10	925	0.614
4	4500	0.12	0.10	925	0.613
5	4520	0.12	0.11	924	0.612
6	4618	0.12	0.10	924	0.61
7	4500	0.12	0.12	924	0.61
8	4503	0.13	0.10	924	0.609
9	4665	0.12	0.10	924	0.607
10	4500	0.13	0.10	924	0.607
11	4715	0.12	0.10	923	0.604
12	4500	0.13	0.10	923	0.604
13	4500	0.12	0.13	923	0.602
14	4511	0.13	0.10	921	0.595
15	4500	0.12	0.17	919	0.585
16	5036	0.12	0.10	919	0.584
17	4500	0.12	0.23	914	0.555
18	4500	0.17	0.10	910	0.533
19	4500	0.12	0.27	910	0.533
20	6010	0.12	0.10	908	0.521
21	4500	0.12	0.31	906	0.515
22	4500	0.12	0.33	905	0.506
23	4523	0.24	0.10	889	0.424
24	6717	0.12	0.50	862	0.279

#### Cutting parameters optimization for fatigue resistance

In the light of our previous research [28], the superposition of machining induced surface residual compressive stress and alternate load changes the ultimate stress level, average stress and stress ratio on the surface of the workpiece, and then affects the initiation and propagation process of fatigue cracks. The influence of surface work hardening on fatigue resistance is two-sided and moderate degree of work hardening is beneficial for the impediment of shear slip and then provides a strengthened resistance to fatigue crack initiation. However, excessive work hardening will also decrease the surface toughness and improve the sensitivity of surface notch under alternate load. This is detrimental to fatigue resistance because of the influence on crack propagation life. In addition, the microscopic stress concentration, which can affect the rate of fatigue crack propagation significantly, is closely related with the surface defect and the surface topography of ball-end milled surfaces [29].

**Fig 9:**
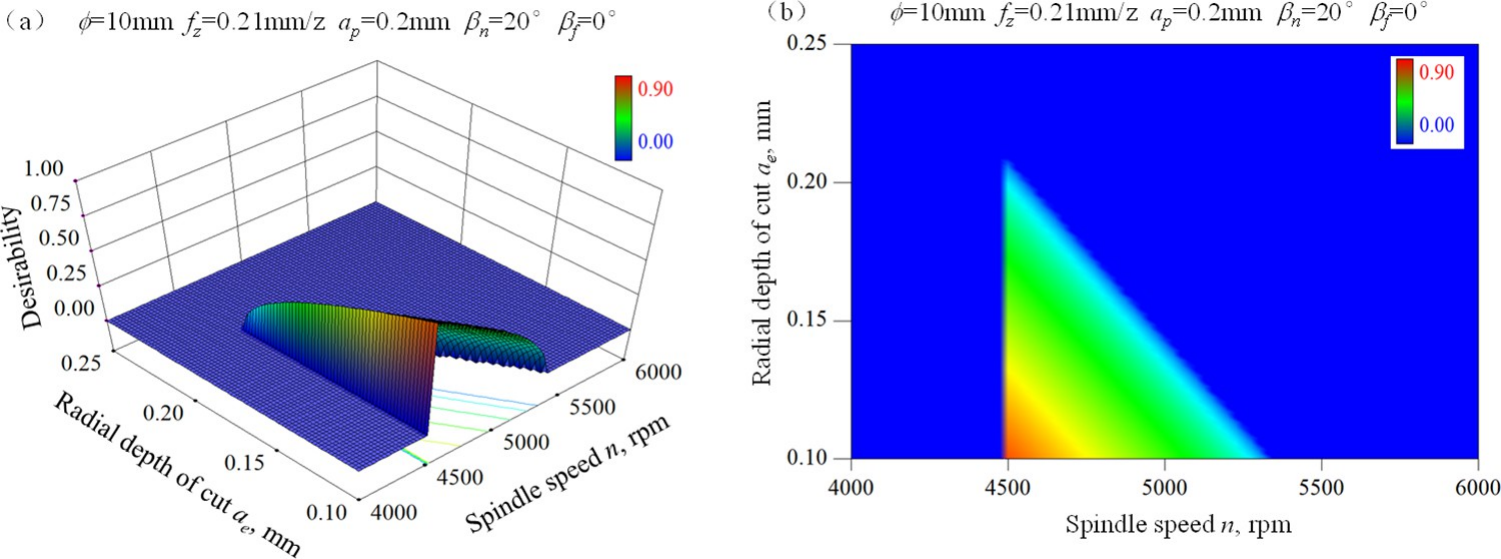
Optimization of cutting parameters for high residual compressive stress in feed direction and microhardness HV 900 purpose, (a) Response surface of desirability, (b) Contour map of desirability.

**Fig 10:**
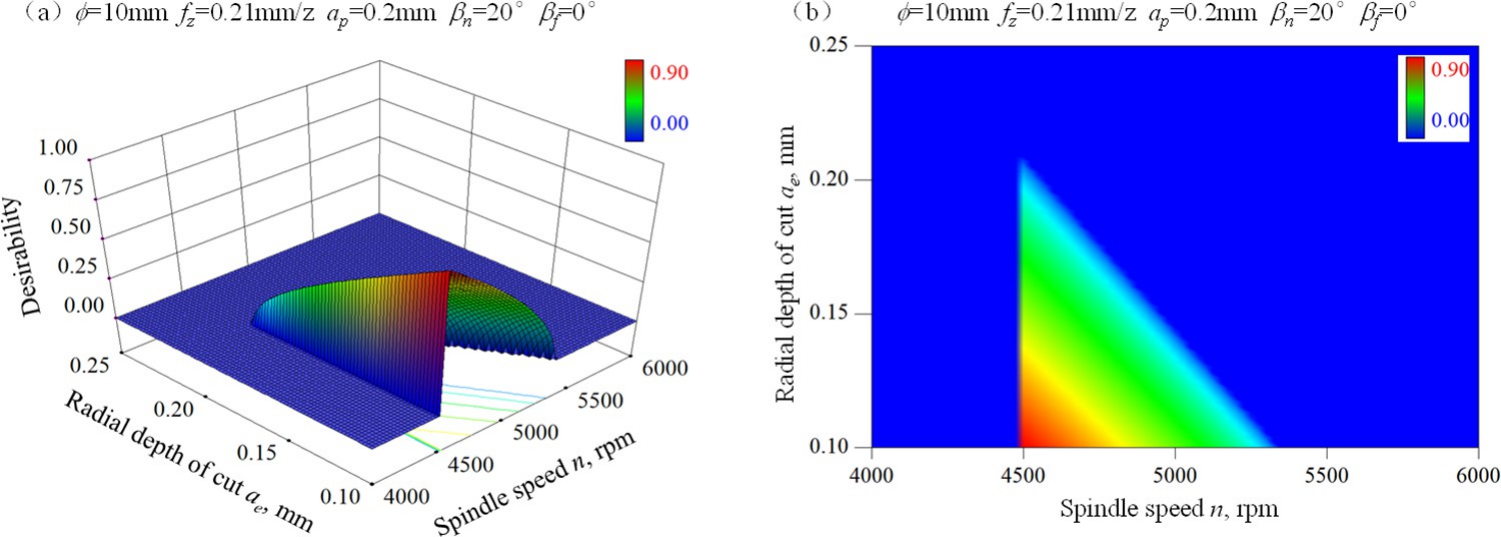
Optimization of cutting parameters for high residual compressive stress in step-over direction and microhardness HV 900 purpose, (a) Response surface of desirability, (b) Contour map of desirability.

Based on the above conclusions, three aspects are considered before optimization of cutting parameters. Firstly, select relatively small radial depth of cut *a*_e_ or large feed per tooth *f*_z_ to decrease the microscopic stress concentration phenomenon. Secondly, choose a high spindle speed *n* or a large feed per tooth *f*_z_ or a large radial depth of cut *a*_*e*_ to ensure the machining efficiency. Lastly, increase the spindle speed *n* or feed per tooth *f*_*z*_ to restrain surface defects. To satisfy the above requirements, the constraints of cutting parameters are set as following: 4500 rpm≤*n*≤8000 rpm, 0.12 mm/z≤*f*_z_≤0.3 mm/z, 0.1 mm≤*a*_e_≤0.3 mm. Furthermore, combined with our previous findings [28], ball-end hard milled surfaces with microhardness approximate HV 900 show better fatigue resistance than the other condition. Then, high residual compressive stress together with surface microhardness close to HV 900 are identified as the optimization goal and the degrees of importance for the two responses is set to be the same. Fig 9 gives the response surface and the contour map of desirability for the goal of high residual compressive stress in feed direction and microhardness HV 900. This situation is defined as case 1. Fig 10 gives the response surface and the contour map of desirability for the goal of high residual compressive stress in step-over direction and microhardness HV 900. This situation is defined as case 2.

Comparing Figs 9 and 10, it can be found that the areas with high desirability for the two cases are very similar. This is because the residual stress in the feed direction and step-over direction has the similar trend with the change of spindle speed and radial depth of cut as shown in Figs 5 and 6. As shown in Figs 9 and 10, the area with high desirability occurs when a low spindle speed and a small radial depth of cut are adopted. The optional range and the maximum value of radial depth of cut decrease with the increase of spindle speed. Similarly, the optional range and the maximum value of spindle speed decrease with the increase of radial depth of cut. The optimization scheme of cutting parameters for fatigue resistance purpose considering the two aforementioned cases is given in Tables 12 and 13, respectively. In actual production, the optimization scheme should be chosen based on comprehensive analysis of the requirement of surface roughness, the alternate load direction, the metal removal rate and so on.

**Table 12:** Optimization scheme of cutting parameters for case 1.

No.	Spindle speed *n*, rpm	Feed per tooth *f*_z_, mm/z	Radial depth of cut *a*_*e*_, mm	*σ*_*feed*_, MPa	HV	Desirability
1	4500	0.21	0.10	-424	900	0.829
2	4593	0.20	0.10	-412	900	0.823
3	4637	0.20	0.10	-407	900	0.819
4	4500	0.20	0.11	-411	900	0.816
5	4500	0.20	0.12	-399	900	0.815
6	4500	0.20	0.12	-396	900	0.813
7	4500	0.19	0.15	-369	900	0.798
8	5007	0.18	0.10	-364	900	0.795
9	5253	0.18	0.10	-339	900	0.779
10	4507	0.18	0.19	-320	900	0.768
11	4500	0.17	0.21	-309	900	0.761
12	4500	0.20	0.10	-423	902	0.761
13	4500	0.16	0.25	-270	900	0.737
14	6031	0.14	0.10	-270	900	0.736
15	4500	0.16	0.26	-266	900	0.734
16	4500	0.15	0.29	-243	900	0.716
17	6481	0.13	0.10	-238	900	0.716
18	4504	0.14	0.30	-237	900	0.715
19	5088	0.12	0.30	-189	900	0.683

**Table 13:** Optimization scheme of cutting parameters for case 2.

No.	Spindle speed *n*, rpm	Feed per tooth *f*_*z*_, mm/z	Radial depth of cut *a*_*e*_, mm	*σ*_*cross-feed*_, MPa	HV	Desirability
1	4500	0.21	0.10	-824	900	0.904
2	4589	0.20	0.10	-816	900	0.901
3	4501	0.20	0.10	-811	900	0.900
4	4680	0.20	0.10	-807	900	0.898
5	4501	0.20	0.11	-783	900	0.890
6	5194	0.18	0.10	-757	900	0.881
7	4500	0.21	0.10	-824	899	0.868
8	5603	0.16	0.10	-715	900	0.866
9	5942	0.15	0.10	-680	900	0.854
10	6052	0.14	0.10	-668	900	0.849
11	6190	0.14	0.10	-653	900	0.844
12	4500	0.19	0.15	-637	900	0.838
13	6366	0.13	0.10	-634	900	0.837
14	6441	0.13	0.10	-625	900	0.834
15	6617	0.12	0.10	-606	900	0.826
16	4500	0.18	0.19	-499	900	0.785
17	4500	0.18	0.20	-468	900	0.772
18	4500	0.17	0.20	-459	900	0.769
19	4500	0.17	0.21	-428	900	0.750
20	4500	0.16	0.26	-310	900	0.703
21	4500	0.16	0.24	-351	899	0.697
22	4500	0.15	0.29	-259	900	0.679
23	4938	0.13	0.30	-182	900	0.641
24	5087	0.12	0.30	-162	900	0.631
25	7473	0.12	0.10	-519	890	0.557

In the future, the physical mechanism during the machining of AISI D2 steel should be further investigated to better explain the relationship between cutting parameters and surface integrity. In addition, the influence of tilt angle and lead angle on wear resistance and fatigue resistance should also be revealed.

## Conclusions

During the manufacturing process of dies or molds with large size and complex curved surfaces, ball-end milling operation is usually used as the final process. The influence mechanism of cutting parameters on surface integrity and the optimization scheme from surface integrity, wear resistance and fatigue resistance perspective were studied during ball-end hard milling process of AISI D2 steel. In this study, the range of cutting speed, feed per tooth and radial depth of cut are 63 m/min to 251 m/min, 0.06 mm/z to 0.3 mm/z and 0.1 mm to 0.5 mm, respectively. Taken together, the main findings in this study can be drawn as following:

1. Radial depth of cut with percent contribution ratio (PCR) 62.05% is the most significant factor influencing surface roughness, followed by spindle speed 13.25% and feed per tooth 6.63%. Surface roughness decreases with the increase of spindle speed, but this effect is the least obvious. The combination of small radial depth of cut and high spindle speed contributes to good surface finish and a satisfying material removal rate simultaneously.
2. Severe work hardening occurred on ball-end milled surface of hardened AISI D2 steel, especially for the low spindle speed condition. The work hardening degree was raised from 12.5% to 38.4% when spindle speed changed from *n* = 8000 rpm to *n* = 2000 rpm for *a*_e_ = 0.1 mm, *a*_p_ = 0.2 mm and *f*_z_ = 0.18 mm/z condition. Moreover, the influence of radial depth of cut on surface microhardness is not obvious when a high spindle speed is adopted. The work hardening degree changed from 12.9% to 15.7% when spindle speed radial depth of cut increases from 0.1 mm to 0.5 mm. Feed per tooth (PCR 25.14%) and spindle speed (PCR 24.79%) are the most influential factor on surface microhardness relatively. The microhardness of machined surface can be improved significantly by selecting small spindle speed and small radial depth of cut during finish machining process.
3. Spindle speed and radial depth of cut are the most significant factor influencing residual stress. The PCR of spindle speed and radial depth of cut reached 73.47% and 18.63% for residual stress in feed direction, 47.11% and 37.51% for residual stress in step-over direction, respectively. High residual compressive stress can be generated by using low spindle speed and low radial depth of cut combination during ball-end milling process. This is mainly because the squeeze between the ball-end milling cutter and workpiece was aggravated. Conversely, the combination of high spindle speed and large radial depth of cut can bring about residual tensile stress due to the obviously increased cutting temperature.
4. Too small feed per tooth or too small radial depth of cut should be avoided from wear resistance point of view during ball-end milling process. This is because though the surface microhardness can be improved, the surface quality will also be deteriorated which is detrimental to wear resistance. Overall, the combination of relatively high spindle speed, small feed per tooth together with small radial depth of cut can meet the wear resistance and the machining efficiency requirement.
5. Cutting parameters can influence the formation of surface defects, the state of residual stress and the degree of microscopic stress concentration. It is unrealistic to ascertain the best cutting parameter combination to satisfy all requirements at the same time and then improve the fatigue resistance of ball-end milled surface. Taken together, the material removal rate and the fatigue resistance can be obtained by using medium-sized cutting parameter combination. Moreover, the optional range and the maximum value of radial depth of cut decrease with the increase of spindle speed.
6. The proposed optimization scheme from surface integrity, wear resistance and fatigue resistance perspective respectively in this study can be used to guide the selection of cutting parameters during ball-end milling of hardened AISI D2 steel for dies/molds manufacturing industries.

## Supporting information

S1 Data (PDF)

S1 File (PDF)

## Abbreviations

*n*: Spindle speed
*f*_z_: Feed per tooth
*a*_e_: Radial depth of cut
*a*_p_: Axial depth of cut
*β*_f_: Lead angle
*β*_n_: Tilt angle
*ϕ*: Diameter of ball-end milling cutter
*γ*: Rake angle Clearance angle
*α*: Clearance angle
*β*: Helix angle
*Z*: Tooth number of ball-end milling cutter
Ra: Surface roughness
HV: Microhardness
*σ_feed_*: Residual stress in feed direction
*σ_cross-feed_*: Residual stress in step-over direction
