# Supplementary material for: Surface integrity optimization for ball-end hard milling of AISI D2 steel based on response surface methodology
Source: PLoS One. 2023 Aug 25;18(8):e0290760. doi: 10.1371/journal.pone.0290760 (PMC10456152; doi:10.1371/journal.pone.0290760)
Supplement: S1 Data — (PDF) [file pone.0290760.s001.pdf]

S1 Data

The average value and standard deviation (St. d.) of surface roughness, microhardness and residual stress is shown in Table A. Surface roughness of milled surfaces was obtained by using white light interferometer Veeco NT9300. Surface microhardness was measured through microhardness tester MH-6. The dwell time and the indentation load were set as 10 s and 0.1 kg, respectively. Residual stress was measured by using X-stress 3000 in the light of $\sin^2\psi$ technique.

Table A. Average value and standard deviation **of surface roughness, microhardness and residual stress.**

No	Spindle speed *n*, rpm	Cutting speed v_c, m/min	Feed per tooth f_z, mm/z	Radial depth of cut a_e, mm	Axial depth of cut a_p, mm	Surface roughness Ra μm	St. d. μm	HV	St. d. HV	Residual stress in FD Mpa	St. d. Mpa	Residual stress in CFD Mpa	St. d. Mpa
1	8000	251	0.3	0.3	0.2	0.49	0.006	810	38	90	12.3	103	13.6
2	8000	251	0.18	0.5	0.2	0.89	0.018	833	39	305	20.8	368	21.1
3	5000	157	0.18	0.3	0.2	0.57	0.009	870	30	-225	14.6	-153	13.8
4	8000	251	0.18	0.1	0.2	0.53	0.022	813	34	-47	8.6	-511	21.1
5	5000	157	0.18	0.3	0.2	0.78	0.023	870	29	-211	12.7	-147	15.2
6	2000	63	0.06	0.3	0.2	1.23	0.032	929	33	-687	8.2	-750	25.6
7	5000	157	0.3	0.1	0.2	0.43	0.002	897	41	-440	16.1	-788	9.3
8	5000	157	0.06	0.5	0.2	1.25	0.010	965	45	-155	13.3	-140	13.1
9	2000	63	0.18	0.5	0.2	1.53	0.010	839	20	-399	17.9	-595	12.1
10	5000	157	0.18	0.3	0.2	0.90	0.010	870	19	-221	16.7	-212	21.5
11	2000	63	0.3	0.3	0.2	0.78	0.010	874	30	-615	15.8	-484	17.7
12	5000	157	0.06	0.1	0.2	0.51	0.013	936	37	-512	15.2	-810	18.4
13	2000	63	0.18	0.1	0.2	0.36	0.016	997	29	-755	23.7	-1128	32.3
14	8000	251	0.06	0.3	0.2	0.66	0.016	897	26	18	9.2	250	16.1
15	5000	157	0.3	0.5	0.2	1.03	0.040	858	30	-84	13.4	-44	20.7
16	5000	157	0.18	0.3	0.2	0.89	0.013	870	31	-40	11.0	-115	16.1
17	5000	157	0.18	0.3	0.2	0.90	0.013	870	23	-76	13.5	-148	13.7
