# Supplementary material for: Surface integrity optimization for ball-end hard milling of AISI D2 steel based on response surface methodology
Source: PLoS One. 2023 Aug 25;18(8):e0290760. doi: 10.1371/journal.pone.0290760 (PMC10456152; doi:10.1371/journal.pone.0290760)
Supplement: S1 File — (PDF) [file pone.0290760.s002.pdf]

S2 File

The Design Expert 8.0 software was used to design and analyze the response surface experiment in this study. The Equation (1) to (4) and the Table 4 to Table 13 shown in this study were obtained based on the software.
